# Screening and Probiotic Potential Evaluation of Bacteriocin-Producing *Lactiplantibacillus plantarum* In Vitro

**DOI:** 10.3390/foods11111575

**Published:** 2022-05-27

**Authors:** Yushan Bu, Yisuo Liu, Yinxue Liu, Shaolei Wang, Qiqi Liu, Haining Hao, Huaxi Yi

**Affiliations:** College of Food Science and Engineering, Ocean University of China, Qingdao 266000, China; bys19941003@163.com (Y.B.); liuyisuo@stu.ouc.edu.cn (Y.L.); liuyinxue_1121@126.com (Y.L.); wangshaolei@stu.ouc.edu.cn (S.W.); liuqiqi9306@126.com (Q.L.); haohaining1994@163.com (H.H.)

**Keywords:** probiotics, bacteriocins, *L. plantarum*, gastrointestinal tolerance, anti-inflammatory activity

## Abstract

Probiotics are gaining attention due to their functions of regulating the intestinal barrier and promoting human health. The production of bacteriocins is one of the important factors for probiotics to exert beneficial properties. This study aimed to screen bacteriocin-producing *Lactiplantibacillus plantarum* and evaluate the probiotic potential in vitro. It was found that *L. plantarum* Q7, *L. plantarum* F3-2 and *L. plantarum* YRL45 could produce bacteriocins and inhibit common intestinal pathogens. These three strains had probiotic potential with tolerance to the gastrointestinal environmental and colonization in the gut, and exhibited various degrees of anti-inflammatory activity and tight junction function in the intestinal barrier. Particularly, *L. plantarum* YRL45 could significantly (*p* < 0.05) reduce the increase in nitric oxide (NO), prostaglandin E2 (PGE2), necrosis factor-α (TNF-α) and interleukin-1β (IL-1β) induced by lipopolysaccharide (LPS), thereby easing inflammatory response. *L. plantarum* F3-2 could remarkably (*p* < 0.05) up-regulate the expression levels of *ZO-1*, *Occludin* and *Claudin-1* in intestinal epithelial injured cells, which was conducive to protecting the intestinal barrier. These findings provided fundamental information about the probiotic properties of bacteriocin-producing *L. plantarum*, which suggested that *L. plantarum* Q7, *L. plantarum* F3-2 and *L. plantarum* YRL45 had the potential to be used as novel probiotic strains.

## 1. Introduction

Probiotics are kinds of living microorganisms, which can be administered in adequate amounts and confer health benefits on the host, such as protecting the intestinal barrier, regulating intestinal flora, alleviating inflammation response and improving stress status [1,2]. Probiotics producing antibacterial substances is one of important factors in regulating intestinal microecology and maintaining host health [3]. The ability to produce antibacterial substances has gradually become an essential evaluation index for screening probiotics. The antibacterial substances from probiotics include organic acids, hydrogen peroxide, bacteriocins, etc. [4]. Bacteriocins are polypeptides or proteins with antibacterial activity synthesized by bacteria during growth, which are mainly used as biological preservatives to control microbial contamination in the food industry [5]. Compared with antibiotics, most bacteriocins of lactic acid bacteria (LAB) have the advantages of safety, high efficiency and no drug resistance, so they have become research hotspots in the field of food preservation and alternatives to antibiotics [6]. In recent years, studies have shown that bacteriocins could also prevent pathogenic infection, regulate the host immune system, maintain intestinal flora balance and reduce inflammatory reaction [7,8,9,10,11]. Huang et al. [12] pointed out that bacteriocins could modulate cytokine levels by regulating Toll-like receptor (TLR), nuclear factor kappa-B (NF-κB) and mitogen-activated protein kinase (MAPK) signaling pathways, and inhibit inflammatory effects caused by pathogenic bacteria or other irritants. Bacteriocins also suppressed infection-induced inflammation and pathogens migration by increasing tight junction proteins (TJPs) expression to strengthen the intestinal barrier [12]. With the wide application of bacteriocin-producing LAB in all kinds of foods, it is significant to explore the probiotic properties of such strains, which will broaden their application prospects and develop new functional foods based on bacteriocin-producing probiotics.

*L. plantarum* are common types of LAB with probiotic effects on the human body, some of which can inhibit pathogenic bacteria, regulate immunity and maintain intestinal health [13,14]. The function and utilization of *L. plantarum* have received much attention. At present, more and more bacteriocin-producing *L. plantarum* have been reported [15,16], but relevant studies were mainly focused on the isolation and purification, antibacterial mechanism and high-efficiency expression of bacteriocins [17,18]. There were few reports on the probiotic functions of bacteriocin-producing *L. plantarum*. Therefore, it is necessary to evaluate the probiotic properties of bacteriocin-producing *L. plantarum*. In this study, bacteriocin-producing *L. plantarum* were screened using the agar diffusion method. The gastrointestinal environmental tolerance and colonization ability of bacteriocin-producing *L. plantarum* in the gut were measured by the method of in-vitro simulation, and the anti-inflammatory activity and tight junction intestinal barrier function were explored using cell models. This study will help to broaden the functional features of bacteriocin-producing LAB and develop them as probiotics.

## 2. Materials and Methods

### 2.1. Strains and Growth Conditions

The 79 strains of *L. plantarum* were isolated from various fermented products and infant feces, which were inoculated into MRS broth (Qingdao Hopebio Technology, Shandong, China) at a proportion of 2% (*v*/*v*) and cultured at 37 °C for 24 h. *Listeria monocytogenes, Escherichia coli*, *Salmonella typhimurium*, *Shigella sonnei* and *Staphylococcus aureus* were used as indicator strains in bacteriostatic experiments. *L. monocytogenes* was grown in BHI medium (Qingdao Hopebio Technology, Shandong, China) at 37 °C for 24 h, and the other four indicator strains were cultivated in LB broth (Qingdao Hopebio Technology, Shandong, China) with shaking at 37 °C for 24 h.

### 2.2. Screening of Bacteriocin-Producing L. plantarum

*L. plantarum* strains were centrifuged (TG20KR-D, Changsha Dongwang, China) at 6500× *g* for 10 min, and the cells were discarded to retain the supernatants. Part of the supernatants were adjusted to pH 6.0 with NaOH to eliminate organic acids interference, and the remaining supernatants were maintained at the original pH, both of which were filtered through 0.22 µm membranes. The agar well diffusion method with BHI semi-solid medium was carried out to determine the antimicrobial activity of *L. plantarum* using *L. monocytogenes* as the indicator strain [19]. Then the *L. plantarum* supernatants with antibacterial activity after excluding organic acids were adjusted to the optimum pH for catalase (5000 U/mg, Solarbio, Beijing, China), papain (800 U/mg, Solarbio, Beijing, China), bromelain (600 U/mg, Solarbio, Beijing, China), trypsin (250 U/mg, Solarbio, Beijing, China), pepsin (250 U/mg, Solarbio, Beijing, China) and proteinase K (30 U/mg, Solarbio, Beijing, China). The strain supernatants were mixed with 1 mg/mL of above six enzymes at 9:1 (*v*/*v*) and incubated at 37 °C for 1 h, respectively, followed by water bath at 70 °C for 5 min. The inhibition zone diameter was determined by agar well diffusion method and the titer was measured by double-dilution method.

### 2.3. Antibacterial Activity against Common Intestinal Pathogens

The supernatants of bacteriocin-producing *L. plantarum* determined in 2.2 were adjusted to pH 6.0 and mixed with the culture medium (BHI or LB broth) of indicator bacteria *L. monocytogenes*, *E. coli*, *S. typhimurium*, *S. sonnei* and *S. aureus* at a ratio of 1:1 (*v*/*v*). The indicator bacteria were inoculated into the mixture mentioned above at 2% (*v*/*v*). The strain supernatants were replaced with MRS broth in control group. The samples were cultured at 37 °C for 25 h, and the absorbance was measured at a wavelength of 600 nm with microplate reader (Multiskan FC, Thermo Fisher Scientific, Waltham, MA, USA) every 5 h to screen out bacteriocin-producing *L. plantarum* that could inhibit common intestinal pathogenic bacteria.

### 2.4. Acid and Bile Salt Tolerance

The acid and bile salt tolerance of *L. plantarum* strains were determined according to the report of Reuben et al. [20] with some modifications. The culture of *L. plantarum* strains were centrifuged at 3700× *g* for 10 min, and supernatants were discarded to collect bacterial cells. The cells were washed twice with phosphate-buffered saline (PBS) and resuspended in PBS with pH 3.0 for 3 h and 0.3% bile salt (Solarbio, Beijing, China) for 5 h, respectively. The viable counts of strains were assayed by gradient dilution method. The survival rate was defined as follows:

(1) $$\mathrm{Survival}\;\mathrm{rate}\;=\frac{{\mathrm{lg}\;N}_{t}}{{\mathrm{lg}\;N}_{0}}\times 100\%$$

N_t_: the number of viable counts after acid or bile salt treatment; N_0_: the number of viable counts before treatment.

### 2.5. Gastric and Intestinal Juice Tolerance

The resistance of strains to gastric and intestinal juice was measured as described by Wang et al. [21] with minor modifications. The cells of strains were collected and resuspended in simulated gastric juice (pH 3.0, containing 3 g/L pepsin). After incubation at 37 °C for 3 h, the cultures were centrifuged and resuspended in simulated intestinal juice (pH 8.0, containing 1 g/L trypsin) for 8 h. The bacterial survival rate was calculated according to plate colony counting method.

(2) $$\mathrm{Survival}\;\mathrm{rate}\;=\frac{{\mathrm{lg}\;N}_{t}}{{\mathrm{lg}\;N}_{0}}\times 100\%$$

N_t_: the number of viable counts after gastric or intestinal juice treatment; N_0_: the number of viable counts before treatment.

### 2.6. Hydrophobicity Assay

Hydrophobicity assay was conducted as previously described with modifications [22]. The bacterial cells were collected, and the absorbance of bacterial solution was adjusted to 0.5 at 600 nm with PBS. A total of 3 mL bacterial solution was mixed with 1 mL of xylene and chloroform, respectively. After oscillation for 2 min, stratification was observed at room temperature by standing for 30 min, which was allowed to separate into two phases. The absorbance of water phase was measured at 600 nm. The hydrophobicity rate was calculated by the formula below:

(3) $$\mathrm{Hydrophobicity}\;\mathrm{rate}\;=\left(1-\frac{A_{t}}{A_{0}}\right)\times 100\%$$

A_t_: the absorbance of aqueous phase; A_0_: the initial absorbance of 0.5.

### 2.7. Self-Aggregation Assay

Self-aggregation was examined using the method of Chen et al. [23] with slight modifications. The bacteria were collected by centrifugation, resuspended with PBS and adjusted to the absorbance of 1.0 at 600 nm. Exactly 4 mL of bacterial suspension was vortexed for 2 min and left for 1 h, 3 h and 5 h at room temperature, respectively. The self-aggregation rate was assessed by measuring the absorbance of upper solution after standing at a wavelength of 600 nm.

(4) $$\mathrm{Self}-\mathrm{aggregation}\;\mathrm{rate}\;=\left(1-\frac{A_{t}}{A_{0}}\;\right)\times 100\%$$

A_t_: the absorbance of upper solution; A_0_: the initial absorbance of 1.0.

### 2.8. Cytotoxic Activity Determination

Raw264.7 and Caco-2 cells were purchased from the Cell Bank of Chinese Academy of Sciences (Shanghai, China), which were grown in high-glucose Dulbecco’s Modified Eagle Medium (DMEM) (Solarbio, Beijing, China), containing 10% (*v*/*v*) fetal bovine serum (FBS) (Biological Industries, Israel) and 1% (*v*/*v*) penicillin/streptomycin (Solarbio, Beijing, China) under an atmosphere of 5% CO_2_ at 37 °C. RAW264.7 and Caco-2 cells were incubated in 96-well plates (2 × 10^4^ cells/well), respectively, and *L. plantarum* strains dispersed in DMEM without FBS and antibiotics at a concentration of 1 × 10^7^ CFU/mL were added to plates. After intervention for 24 h, 10 µL of CCK-8 (GlpBio, Shanghai, China) was added into wells and incubated at 37 °C for 2 h, and the absorbance was measured at 450 nm [24].

### 2.9. Anti-Inflammatory Activity Detection

RAW264.7 cells were seeded in 24-well plates (2 × 10^5^ cells/well), which were treated with LPS (1 μg/mL) as well as *L. plantarum* strains (1 × 10^7^ CFU/mL) for 24 h. The cell supernatants were taken to determine the secretion of NO using a NO assay kit (Nanjing Jiancheng Bio, Nanjing, China), and PGE2, TNF-α, IL-6, IL-1β and IL-10 in supernatants were quantified using enzyme-linked immunosorbent assays (ELISA) kit (Suzhou Calvin Bio, Suzhou, China).

### 2.10. Real-Time Quantitative Polymerase Chain Reaction (RT-qPCR)

Caco-2 cells were plated at a density of 1 × 10^6^ cells/well in 6-well plates, and cells were incubated with LPS (10 μg/mL) and *L. plantarum* strains (1 × 10^7^ CFU/mL) for 24 h. Total RNA was extracted from Caco-2 cells using TRNzol Universal (Tiangen, Beijing, China) and RNA was reverse transcribed into cDNA using ReverTra Ace qPCR RT Master Mix with gDNA Remover (Toyobo, Osaka, Japan). RT-qPCR was carried out on CFX96 RealTime PCR System (Bio-rad, Hercules, CA, USA) with 2 × SYBR Green Realtime PCR Master Mix (Toyobo, Osaka, Japan) in a total volume of 25 μL. GAPDH was used as an internal control and gene expression was converted to relative fold differences by 2^−ΔΔCt^ method [25]. Primers were synthesized by Shanghai Sangon Biotech and the primer sequences were listed in Table 1.

### 2.11. Statistical Analysis

All experiments were repeated three times and the data were shown as mean ± standard deviation (SD) (*n* = 3) in tables and figures. IBM SPSS Statistics 22.0 was used for statistical analysis. The significance was analyzed by independent sample *t*-test between two groups and one-way analysis of variance (ANOVA) and Duncan’s test for multiple comparisons at *p* < 0.05.

## 3. Results and Discussion

### 3.1. Screening of Bacteriocin-Producing L. plantarum

Most of the bacteriocins of LAB are divided into two classes, including Class I and Class II [6]. Class II bacteriocins are prospective and widely studied. The high specificity of class II bacteriocins against *L. monocytogenes*, a typical characteristic of class II bacteriocins [26], has meant that this group has attracted much attention. In this study, *L. monocytogenes* was used as indicator bacteria for preliminary screening, and 18 strains showing antibacterial activity were screened from 79 strains of *L. plantarum* (Table 2). *L. plantarum* (CXG-2, HZPS-2, JLSC1-2, CXG-8, CXG-6, NDF1000S-4, JLSC2-4, JNS-3, SXLJ-2, HZZC-2, HCG4-2, SHG-1, CXG-4 and HZPS-1) showed an antagonistic effect against *L. monocytogenes,* only when organic acids were not excluded, indicating that acids derived from those strains could be responsible for antibacterial activity. *L. plantarum* Q7, *L. plantarum* F3-2, *L. plantarum* YRL45 and *L. plantarum* JLSC2-5 could produce antibacterial activity before and after the removal of acids, which suggested that bacterial metabolites, including hydrogen peroxide or bacteriocins, might work.

**Figure 1:**
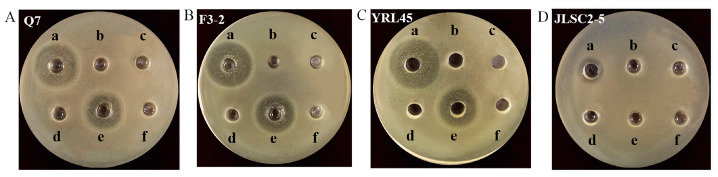
Effect of proteases on antibacterial activity of *L. plantarum* Q7 (**A**), *L. plantarum* F3-2 (**B**), *L. plantarum* YRL45 (**C**) and *L. plantarum* JLSC2-5 (**D**). (a) Without protease treatment. (b) Papain treatment. (c) Bromelain treatment. (d) Trypsin treatment. (e) Pepsin treatment. (f) Proteinase K treatment.

When catalase was added to the supernatants of *L. plantarum* Q7, *L. plantarum* F3-2, *L. plantarum* YRL45 and *L. plantarum* JLSC2-5, it turned out that the supernatants of four strains could exert bacteriostatic activity without significant difference, before and after the addition of catalase (*p* > 0.05) (Table 3), illustrating that the bacteriostatic characteristic of four strains was not attributed to hydrogen peroxide. After putting proteases into the supernatant of *L. plantarum* Q7, *L. plantarum* F3-2, *L. plantarum* YRL45 and *L. plantarum* JLSC2-5, it could be seen from Figure 1 that the four strains were deprived of their antibacterial capacity under the action of papain, bromelain, trypsin and protease K. In addition, the incubation of supernatants with pepsin led to the decreased inhibitory activity of *L. plantarum* Q7, *L. plantarum* F3-2 and *L. plantarum* YRL45 and lost bacteriostatic activity of *L. plantarum* JLSC2-5, which demonstrated that the antibacterial products of the four strains might be polypeptides or proteins that would be degraded by various proteases to different degrees. Therefore, the antagonistic effect produced by *L. plantarum* Q7, *L. plantarum* F3-2, *L. plantarum* YRL45 and *L. plantarum* JLSC2-5 was achieved by the secretion of bacteriocins.

### 3.2. Antibacterial Activity of Bacteriocin-Producing L. plantarum against Common Intestinal Pathogens

**Figure 2:**
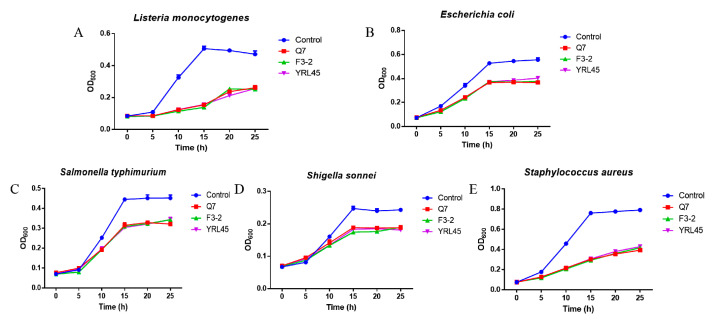
Antibacterial activity of *L. plantarum* Q7, *L. plantarum* F3-2 and *L. plantarum* YRL45 against *L. monocytogenes* (**A**), *E. coli* (**B**), *S. typhimurium* (**C**), *S. sonnei* (**D**) and *S. aureus* (**E**).

It was reported that the bacteriocin-producing LAB had a certain impact on the host intestinal flora [21,26], so it was essential to explore the effect of bacteriocin-producing *L. plantarum* on common intestinal pathogens. *E. coli*, *S. typhimurium*, *S. sonnei* and *S. aureus* are common intestinal pathogenic bacteria, which can easily result in bodily infections, promote intestinal inflammatory reaction and destroy the intestinal barrier, bringing about diarrhea, colitis and other diseases [27,28]. The supernatants of *L. plantarum* Q7, *L. plantarum* F3-2, *L. plantarum* YRL45 and *L. plantarum* JLSC2-5 were co-cultured with enteropathogens, and the results in Figure 2 revealed that besides *L. monocytogenes*, *L. plantarum* Q7, *L. plantarum* F3-2 and *L. plantarum* YRL45 also showed inhibitory activity against *E. coli*, *S. typhimurium*, *S. sonnei* and *S. aureus*. Hence, the three strains had the potential to resist the infection of common pathogens in the gastrointestinal tract and were selected for subsequent experiments.

### 3.3. Tolerance of Bacteriocin-Producing L. plantarum to Acid and Bile Salt

Though the acid in the stomach and the bile salt in the intestine can prevent the contamination of external harmful microorganisms, they affect the survival of exogenous probiotics in the gastrointestinal tract. Therefore, the tolerance of probiotics to acid and bile salt is a prerequisite for their efficacies in vivo. The pH of human gastric juice is generally maintained at about 3.0, and the bile salt concentration in the small intestine is 0.3% on average. As shown in Table 4, *L. plantarum* Q7, *L. plantarum* F3-2 and *L. plantarum* YRL45 showed good acid tolerance ability after 3 h of incubation with pH 3.0 gastric acid. The viable counts reached 10^8^ CFU/mL and the survival rate was more than 92%. When *L. plantarum* Q7, *L. plantarum* F3-2 and *L. plantarum* YRL45 were cultured in 0.3% bile salt for 5 h, the number of viable cells was higher than 10^7^ CFU/mL. These three strains had good tolerance to bile salt and the survival rate ranged from 76.37 ± 0.24% to 80.05 ± 0.84%.

### 3.4. Tolerance of Bacteriocin-Producing L. plantarum to Gastric and Intestinal Juice

The tolerance of strains in gastric and intestinal fluid must be considered when selecting potential probiotic strains. Gastric juice and intestinal juice contain pepsin and trypsin, which can hydrolyze the proteins of probiotics cells and have negative influence on the survival of strains. Probiotics are expected to exert beneficial effects when they can remain alive after passing through the gastrointestinal tract. It was observed that *L. plantarum* Q7, *L. plantarum* F3-2 and *L. plantarum* YRL45 had strong resistance to gastric juice, when subjected to simulated gastric juice for 3 h, and the viable counts and survival rate were more than 10^8^ CFU/mL and 92%, respectively (Table 5). After gastric juice treatment, *L. plantarum* Q7, *L. plantarum* F3-2 and *L. plantarum* YRL45 were maintained for 8 h in simulated intestinal juice. It was found that the survival rate was over 98%, which revealed that these three strains could tolerate the gastrointestinal conditions and maintain the relatively high levels of viability when administered orally.

### 3.5. Hydrophobicity and Self-Aggregation Ability of Bacteriocin-Producing L. plantarum

**Figure 3:**
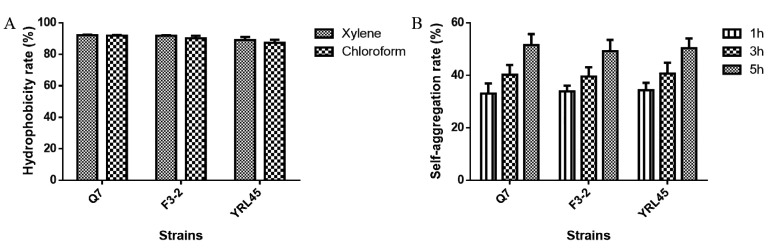
Hydrophobicity (**A**) and self-aggregation (**B**) of *L. plantarum* Q7, *L. plantarum* F3-2 and *L. plantarum* YRL45.

In addition to tolerating the adverse gastrointestinal environment, the probiotic strains must have adhesion capability to colonize in the digestive tract. The hydrophobicity and self-aggregation of strains are closely related to their adhesion ability. Generally, higher hydrophobicity and self-aggregation may favor the adhesion of bacteria to epithelia [29]. It was observed that *L. plantarum* Q7, *L. plantarum* F3-2 and *L. plantarum* YRL45 exhibited excellent hydrophobicity for xylene and chloroform, which was higher than 85% (Figure 3A). As an extension of stewing time, the self-aggregation rate of the three strains was more than 50% after the period of 5 h (Figure 3B). In view of the above observations, *L. plantarum* Q7, *L. plantarum* F3-2 and *L. plantarum* YRL45 could adhere to the intestinal mucosa, which provided the basis for them to exert beneficial functions.

### 3.6. Cytotoxicity of Bacteriocin-Producing L. plantarum

**Figure 4:**
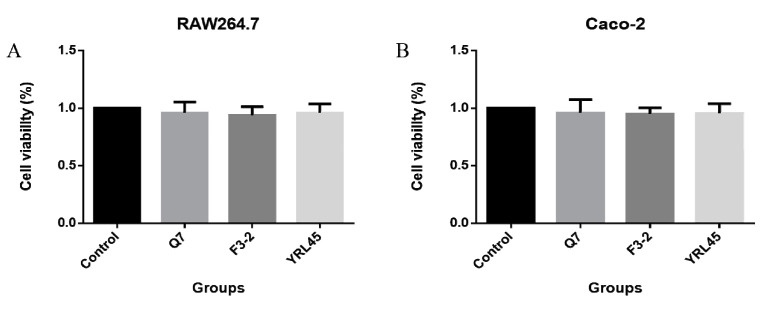
Effect of *L. plantarum* Q7, *L. plantarum* F3-2 and *L. plantarum* YRL45 on RAW264.7 (**A**) and Caco-2 (**B**) cells viability.

The effect of *L. plantarum* Q7, *L. plantarum* F3-2 and *L. plantarum* YRL45 on cell proliferation was evaluated to investigate the cytotoxicity of bacteriocin-producing *L. plantarum*. As was evident from Figure 4, the cell viability of RAW264.7 and Caco-2 cells was more than 93%, and there was no significant difference in the cellular survival rate compared with the control group (*p* > 0.05), when the concentration of strains was 10^7^ CFU/mL, which suggested that the addition of bacteriocin-producing *L. plantarum* Q7, *L. plantarum* F3-2 and *L. plantarum* YRL45 had no negative effect on the growth of RAW264.7 and Caco-2 cells and these three strains could be used for further research.

### 3.7. Anti-Inflammatory Activity of Bacteriocin-Producing L. plantarum

**Figure 5:**
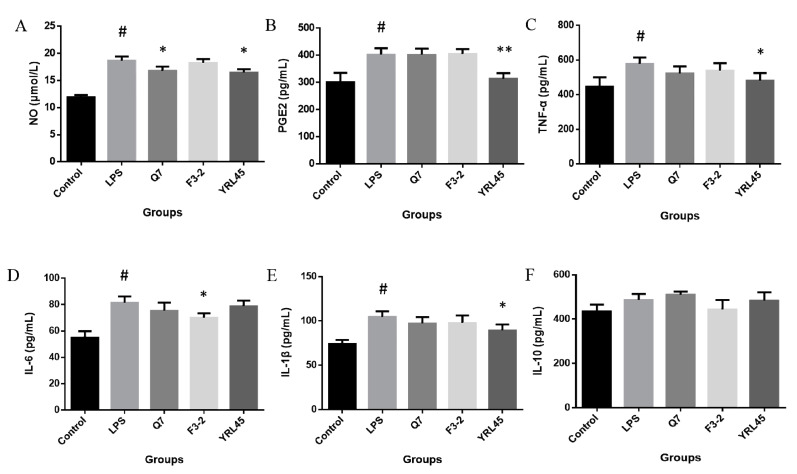
Effect of *L. plantarum* Q7, *L. plantarum* F3-2 and *L. plantarum* YRL45 on the secretion of NO (**A**), PGE2 (**B**), TNF-α (**C**), IL-6 (**D**), IL-1β (**E**) and IL-10 (**F**) in Raw264.7 cells. # *p* < 0.05: LPS group compared with control group. * *p* < 0.05: strain groups compared with LPS group. ** *p* < 0.01: strain groups compared with LPS group.

It was reported that bacteriocin biosynthesis of probiotic *L. plantarum* could contribute to the anti-inflammatory capacity [30]. In order to investigate the anti-inflammatory activity of three bacteriocin-producing *L. plantarum*, RAW264.7 cells induced by LPS were used as the inflammatory model in vitro, and the changes of six inflammatory factors in cells were measured after the intervention of strains. NO is one of the key signaling molecules in inflammation, and excessive NO can cause oxidative injury and inflammatory response [31]. Our results showed that *L. plantarum* Q7 and *L. plantarum* YRL45 significantly suppressed the production of NO induced by LPS in RAW264.7 cells (*p* < 0.05) (Figure 5A), which alleviated the occurrence of inflammation to a certain extent. PGE2 is an important immunomodulatory factor, but the high concentration of PGE2 can exacerbate the inflammatory process [32]. It was found that *L. plantarum* YRL45 exhibited anti-inflammation ability, which was demonstrated by the significant down-regulation of LPS-induced elevation of PGE2 level (*p* < 0.01) (Figure 5B). The pro-inflammatory cytokines TNF-α, IL-6 and IL-1β play critical roles in the progression of inflammation [31]. Treatment with LPS significantly up-regulated the content of TNF-α, IL-6 and IL-1β (*p* < 0.05), whereas *L. plantarum* YRL45 remarkably inhibited the release of TNF-α and IL-1β (*p* < 0.05) (Figure 5C,E), and *L. plantarum* F3-2 showed a substantial inhibition in the enhancement of IL-6 production after inflammatory inducer stimulation (*p* < 0.05) (Figure 5D). IL-10 is a multifunctional cytokine with anti-inflammatory property [33], but the three strains had no significant effect on the level of IL-10 (*p* > 0.05) (Figure 5F). These findings suggested that *L. plantarum* Q7, *L. plantarum* F3-2 and *L. plantarum* YRL45 could reduce the levels of various pro-inflammatory factors to different extents and had certain anti-inflammatory capacity in vitro. Similarly, the anti-inflammatory activity of bacteriocins from LAB has been explored by cell models in previous studies. Nisin is the most widely applied bacteriocin in the food industry. Huang et al. [34] found that Nisin Z isolated from *Lactococcus lactis* inhibited the release of IL-6, IL-1β and TNF-α and increased IL-10 in LPS-induced MCF10A cells. Moreover, Nisin A from *L. lactis* was reported to diminish TNF-α level induced by LPS in peripheral blood mononuclear cells [35]. These results showed that Nisin produced by *L. lactis* had strong anti-inflammatory activity in vitro. Furthermore, Yu et al. [36] investigated the protective effect of bacteriocin MccJ25 on the inflammatory response of IPEC-J2 cells induced by enterotoxigenic *E. coli*. MccJ25 was found to relieve inflammation response through modulation of IL-6, IL-8 and TNF-α levels via inhibition of MAPK and NF-κB activation. At present, it is difficult to separate and purify bacteriocins from strains, and most bacteriocins are susceptible to be degraded by proteases after oral delivery. Bacteriocin-producing probiotics can act as carriers to help bacteriocins colonize and exert probiotic function in the gut [12]. Consequently, it is of great value to carry out research on bacteriocin-producing probiotics in terms of anti-inflammatory activity.

### 3.8. Tight Junction Intestinal Barrier Function of Bacteriocin-Producing L. plantarum

**Figure 6:**
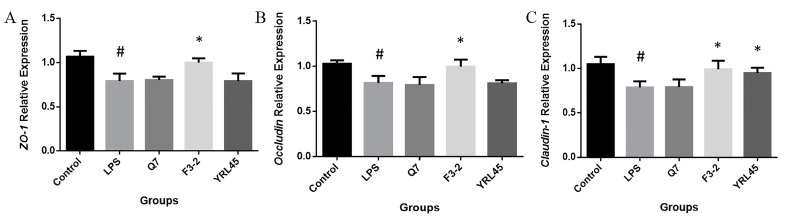
Effect of *L. plantarum* Q7, *L. plantarum* F3-2 and *L. plantarum* YRL45 on the gene expression of *ZO-1* (**A**), *Occludin* (**B**) and *Claudin-1* (**C**) in Caco-2 cells. # *p* < 0.05: LPS group compared with control group. * *p* < 0.05: strain groups compared with LPS group.

TJPs are important forms of intercellular connections and the most vital structures that constitute the mucosal mechanical barrier, composed of structural proteins, such as ZOs, Occludin, Claudins and all kinds of junction protein molecules [37]. It was reported that probiotics could maintain and improve the integrity of the intestinal barrier by regulating the expression of genes related to the tight junction between epithelia [38]. We established a model of intestinal epithelial barrier injury and determined the gene expression levels of *ZO-1*, *Occludin* and *Claudin-1*. The results showed that *L. plantarum* F3-2 up-regulated those genes expression significantly (*p* < 0.05), and *L. plantarum* YRL45 notably elevated the expression of *Claudin-1* (*p* < 0.05) (Figure 6), which inhibited the intestinal barrier damage caused by LPS and presented a function of tight connection with the intestinal barrier, thus, contributing to the protection of the intestinal barrier. A previous study pointed out that bacteriocin separated from a fecal strain of *E. coli* could repair intestinal barrier dysfunction by increasing transepithelial electrical resistance, reducing the release of lactate dehydrogenase and promoting the assembly of Occludin and Claudin-1 in intestinal epithelial impaired cells [36]. Heeney et al. [39] applied bacteriocin plantaricin EF (Pln EF) from *L. plantarum* to Caco-2 cells challenged with TNF-α and interferon-γ (IFN-γ). The combined application of PlnE and PlnF prevented the increase in transcellular permeability and IL-8 level, reflecting that *L. plantarum* bacteriocin could maintain the integrity of the intestinal epithelial barrier in vitro. Our results were consistent with the above studies and provided a basis for the development of bacteriocin-producing *L. plantarum* to play a protective function in the intestinal barrier. In this study, *L. plantarum* Q7, *L. plantarum* F3-2 and *L. plantarum* YRL45 showed functional differences in vitro, and they could be taken as promising candidates for further probiotic function evaluation in vivo.

## 4. Conclusions

In summary, bacteriocin-producing *L. plantarum* Q7, *L. plantarum* F3-2 and *L. plantarum* YRL45 with antibacterial activity against common intestinal pathogens were screened using the agar diffusion method. These three bacteriocin-producing *L. plantarum* had good tolerance to acid, bile salt, gastric and intestinal juice as well as the potential ability to colonize in the gastrointestinal tract, which would be confirmed in vivo. *L. plantarum* Q7, *L. plantarum* F3-2 and *L. plantarum* YRL45 could exert anti-inflammatory effect at varying degrees. *L. plantarum* F3-2 and *L. plantarum* YRL45 protected the intestinal barrier by increasing the expression of TJPs-related genes. Taken overall, *L. plantarum* F3-2 exhibited the best probiotic properties in vitro among these three strains, and further research is required to elucidate the probiotic functions and mechanisms of *L. plantarum* F3-2 in vivo.

## Figures and Tables

**Table 1:** Primer sequences used for RT-qPCR.

Genes	Primer Sequences (5′-3′)
GAPDH	F: AACGGATTTGGTCGTATTG
	R: GCTCCTGGAAGATGGTGAT
ZO-1	F: CGGGACTGTTGGTATTGGCTAGA
	R: GGCCAGGGCCATAGTAAAGTTTG
Occludin	F: GGGCATTGCTCATCCTGAAG
	R: GCCTGTAAGGAGGTGGACTT
Claudin-1	F: GCGCGATATTTCTTCTTGCAGG
	R: TTCGTACCTGGCATTGACTGG

**Table 2:** Screening of *L. plantarum* with antibacterial activity.

Strains	Inhibition Zone Diameter (mm)	
	Acid Not Excluded	Acid Excluded
Q7	26.85 ± 0.40	26.94 ± 0.56
F3-2	27.08 ± 0.51	26.98 ± 0.41
YRL45	26.77 ± 0.22	26.89 ± 0.29
JLSC2-5	13.89 ± 0.39	13.87 ± 0.38
CXG-2	13.30 ± 0.33	7.80
HZPS-2	13.00 ± 0.65	7.80
JLSC1-2	12.97 ± 0.29	7.80
CXG-8	12.91 ± 0.41	7.80
CXG-6	12.49 ± 0.50	7.80
NDF1000S-4	12.41 ± 0.41	7.80
JLSC2-4	12.40 ± 0.43	7.80
JNS-3	12.00 ± 0.33	7.80
SXLJ-2	11.84 ± 0.26	7.80
HZZC-2	11.53 ± 0.47	7.80
HCG4-2	11.47 ± 0.33	7.80
SHG-1	10.87 ± 0.51	7.80
CXG-4	10.77 ± 0.40	7.80
HZPS-1	10.14 ± 0.54	7.80

Note: the outside diameter of Oxford cup was 7.80 mm.

**Table 3:** Effect of catalase on antibacterial activity of *L. plantarum* Q7, *L. plantarum* F3-2, *L. plantarum* YRL45 and *L. plantarum* JLSC2-5.

Strains	Inhibition Zone Diameter (mm)		Titer (AU/mL)
	Without Catalase	With Catalase	With or Without Catalase
Q7	27.03 ± 0.23 ^a^	26.96 ± 0.40 ^a^	400
F3-2	26.95 ± 0.36 ^a^	26.99 ± 0.39 ^a^	400
YRL45	26.95 ± 0.50 ^a^	26.89 ± 0.34 ^a^	400
JLSC2-5	13.93 ± 0.36 ^a^	13.90 ± 0.25 ^a^	50

Note: the same lowercase letter within a line indicated that there was no significant difference (*p* > 0.05).

**Table 4:** Acid and bile salt tolerance of *L. plantarum* Q7, *L. plantarum* F3-2 and *L. plantarum* YRL45.

Strains	Acid (3 h of Incubation)		Bile Salt (5 h of Incubation)	
	Viable Counts (lg CFU/mL)	Survival Rate (%)	Viable Counts (lg CFU/mL)	Survival Rate (%)
Q7	8.71 ± 0.06	93.43 ± 0.16	7.44 ± 0.04	80.05 ± 0.84
F3-2	8.68 ± 0.04	92.82 ± 0.97	7.12 ± 0.03	76.37 ± 0.24
YRL45	8.99 ± 0.07	96.40 ± 0.63	7.29 ± 0.03	78.08 ± 0.28

**Table 5:** Gastric and intestinal juice tolerance of *L. plantarum* Q7, *L. plantarum* F3-2 and *L. plantarum* YRL45.

Strains	Gastric Juice (3 h of Incubation)		Intestinal Juice (8 h of Incubation)	
	Viable Counts (lg CFU/mL)	Survival Rate (%)	Viable Counts (lg CFU/mL)	Survival Rate (%)
Q7	8.75 ± 0.05	93.97 ± 1.04	8.64 ± 0.04	99.02 ± 0.89
F3-2	8.67 ± 0.01	93.04 ± 0.52	8.48 ± 0.07	98.77 ± 0.55
YRL45	8.65 ± 0.06	92.91 ± 1.18	8.52 ± 0.04	98.96 ± 0.95

## Data Availability

The data presented in this study are available on request from the corresponding author.
